# Tracing the aggregation pathway of the scaffold protein DISC1: Structural implications for chronic mental illnesses

**DOI:** 10.1016/j.yjsbx.2025.100128

**Published:** 2025-05-24

**Authors:** Abhishek Cukkemane, Nina Becker, Tatsiana Kupreichyk, Henrike Heise, Dieter Willbold, Oliver H. Weiergräber

**Affiliations:** a Institute of Biological Information Processing (IBI-7: Structural Biochemistry), Forschungszentrum Jülich, Jülich, Germany; b Heinrich Heine University Düsseldorf, Institut für Physikalische Biologie, Düsseldorf, Germany

**Keywords:** Schizophrenia, Disrupted in schizophrenia 1, Aggregation pathway, Proteinopathy, Biophysical analysis, Drug development, Protein-protein interaction

## Abstract

Disrupted in schizophrenia 1 (DISC1) is a pleiotropic scaffold protein that is postulated to comprise large disordered regions and four distinct structured segments with a high proportion of helical or coiled-coil fold. DISC1 associates with over 300 proteins and is associated with several physiological roles ranging from mitosis to cellular differentiation. Yet, the structural features of the protein are poorly characterized. The C-terminal region (C-region, res. 691–836) forms a tetramer and can also aggregate into amyloid-like fibers, potentially linked to schizophrenia and other chronic mental illnesses. Using a combination of biophysical and structural biology applications, we investigate the structural heterogeneity of three mutants of the C-region, viz., the S713E, S704C and L807-frameshift mutants. We provide evidence for the plasticity of the C region; a thin border separates the conformational flexibility of DISC1 required for interaction with a myriad of partners from disruptive aggregation. Snapshots of aggregates and fibrils growing from a nucleus are presented, along with data supporting the role of the minimal fibrillizing element in the C-region, the β-core. This segment also houses a stretch of residues that is critical for the binding of NDEL1 proteins in the mitotic spindle complex and is absent in the non-binding splice variant DISC1Δ22aa. Physiologically, both the splice variant and the fibers represent loss-of-function states that disrupt cellular division. Our findings highlight the need to decipher the structural elements within the DISC1 C-region to comprehend its physiological role and aggregation-related anomalies, and to establish a rationale for drug development.

## Highlights

• DISC1 C-region exhibits structural plasticity, that is essential for function but also carries risk of aggregation.
• Mutations in DISC1 (S713E, S704C, L807-frameshift) are susceptible transitions from functional to aggregated states.
• The potential fibrillizing region β-core overlaps with the NDEL1 binding site, crucial for mitotic spindle function.
• Understand DISC1 structure and the aggregation pathway is essential for understanding its role in chronic mental illness.

## Introduction

Schizophrenia, major depressive disorder (MDD), bipolar disorder (BD) and autism spectrum disorder represent closely associated neurodevelopmental and chronic mental illnesses (CMIs). The aetiological factors that contribute to these disorders remain poorly understood as they involve an interplay of multiple factors, including biological, environmental, and social conditions. A major biological risk factor that was identified about two decades ago in a Scottish family with several severe psychiatric disorders was disrupted in schizophrenia 1 (DISC1) (Blackwood et al., 2001, Millar et al., 2000). DISC1 is a multi-functional hub that regulates the activities of over 300 different enzymes and proteins, including molecules of clinical and therapeutic relevance (Yerabham et al., 2013, Millar et al., 2003). Considering its regulatory significance, DISC1 is involved in a myriad of physiological roles across various cellular functions such as mitosis, proliferation, neuronal development, and synaptogenesis (Bradshaw and Korth, 2019, Tropea et al., 2018, Hikida et al., 2012).

In a recent study, the C-region (Cukkemane et al., 2021) was demonstrated to assemble into a tetramer that represents the functional unit, which associates with Nuclear Distribution Element 1 (NDE1, formerly known as NudE), Nuclear Distribution Element Like 1 (NDEL1, formerly known as Nudel) and platelet activating factor acetylhydrolase 1b regulatory subunit 1 (PAFAH1B1, formerly LIS1) in a cooperative manner in the mitotic spindle complex. Dysfunctional DISC1 aggregates into amyloid-like fibrils that are stained by the thoflavin T (ThT) dye. Using bioinformatics analysis, we identified a pseudo-repeat sequence (res. 717-761) that may represent the scaffold, the β-core of the fibril (Cukkemane et al., 2021). This region encompasses a stretch of amino acids that will herein be referred to as the Δ22 region (748-769). The Δ22 region is absent in the splice variant DISC1^Δ22aa^ (Taylor et al., 2003, Kamiya et al., 2006) (also known as Lv-DISC1 (Nakata et al., 2009), which renders it unable to bind to NDEL1 and PAFAH1B1 proteins, resulting in defective neurite outgrowth in PC12 cells.

**Fig. 1:**
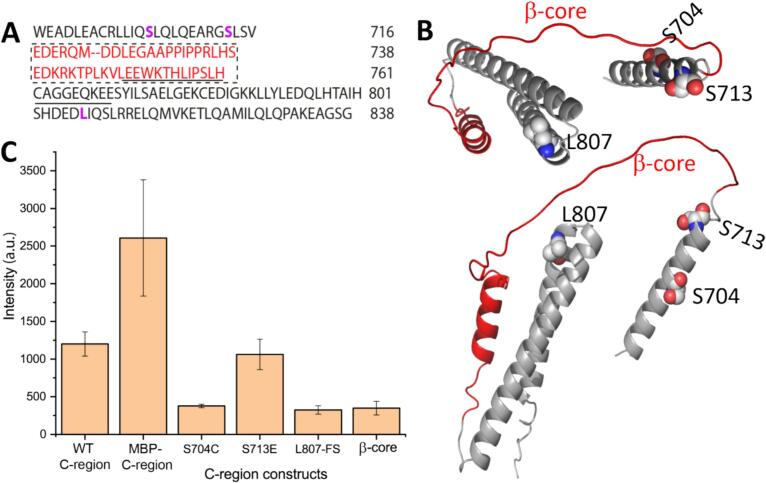
(A) The protein sequence of the DISC1 C-region numbered in the context of the full-length protein, with the pseudo-repeat sequence constituting the β-core (717–761) shown in the dotted box in red; the underlined part corresponds to residues in the Δ22 region (748–769); residues S704, S713, and L807 highlighted in bold pink. (B) Representation of the DISC1 monomeric C‑region model generated using AlphaFold2, featuring two different views. (C) ThT fluorescence assay of the DISC1 C-region, its mutant variants, and the β-core at 25 °C. Experiments for the different proteins (n = 3) were corrected for background by subtracting the buffer control. Bars illustrate mean ± SD. (For interpretation of the references to colour in this figure legend, the reader is referred to the web version of this article.)

Further progress towards understanding the role of the C-region of DISC1 (Fig. 1**A and 1B**) and the significance of the Δ22 region requires delineating the structure–function relationships of the protein from a pathological perspective. Therefore, it is important to understand how mutations within the C-region contribute to the pathophysiology of the various disorders associated with DISC1 dysfunction. In this context, three different C-region variants were selected, *i.e.,* S704C, S713E and the L807-frameshift (L807-FS). The S704C (Leliveld et al., 2009, Leliveld et al., 2008, Narayanan et al., 2011, Hashimoto et al., 2006) mutant forms aggregate deposits in 25 % of post-mortem brain samples from subjects suffering from schizophrenia, BD and MDD. The S713E mutation (Ishizuka et al., 2011) is responsible for Bardet–Biedl syndrome; the Ser residue serves the role of a phosphorylation switch that promotes the transition of neuronal progenitor cells from proliferation to migration during corticogenesis. Finally, the frameshift mutation affecting residues downstream of L807 was first identified in an American family suffering from schizophrenia and schizoaffective disorders and gives rise to aggregated complexes (Sachs et al., 2005).

To comprehend the roles of these residues in DISC1 pathophysiology, it is important to view the function of the protein from a structural perspective. NMR structures of an N-terminally truncated version of the murine C-region (Ye et al., 2017, Wang et al., 2019) highlighted that this region was largely unstructured but adopted helical motifs containing coiled-coil (CC) structure in the presence of a ligand. A typical CC domain is characterized by the presence of heptad repeats (*abcdefg*) in which positions *a* and *d* are occupied by hydrophobic residues and the remaining sites by either polar or charged ones. In these structures, residue L807 is part of the coiled-coil domain. An ab-initio model (Yerabham et al., 2018) of the C-region that is consistent with SAXS data positioned S704 as polar entity in the coiled-coil domain, which also flanks the β-core and S713 present in between two helical coils. These serine residues do not contribute to the oligomeric interface (Cukkemane et al., 2021) and both are accessible to interact with physiological partners. This naturally poses several interesting questions. Firstly, how exactly do mutations within a CC heptad sequence that replace a polar side chain with a small apolar or a charged one, specifically S704C and S713E, affect protein stability? Secondly, does the β-core that houses part of the Δ22 region represent the scaffold of the DISC1 C-region fibrils?

To address these questions and comprehend the impact of point mutations in DISC1 on the pathophysiology of schizophrenia and related CMIs, we employed a combination of biophysical techniques and structural biology applications. Here, we demonstrate that the β-core contains determinants for the aggregation of the C-region. The isolated β-core peptide and the DISC1 C-region mutants form aggregates that may serve as nuclei that are oily droplet-like. These findings are complemented with ThT fluorescence data and thermodynamic parameters of the aggregates formed by the C-region variants. To conclude, we show the individual steps involved in the complexation of the DISC1 C-region into supramolecular entities. These findings extend our previous report (Cukkemane et al., 2021) on the significance of the aggrandization of the DISC1 C-region into fibrillar assemblies from a clinical and pathophysiological perspective and provide insights into therapeutic strategies.

## Experimental procedures

### Protein expression and purification

The DISC1 C-region (WT), C-region fused to maltose-binding protein (MBP-C-region), S704C, S713E, L807-FS and β-core (717-761) proteins were expressed as His_6_ fusion constructs, as described previously (Cukkemane et al., 2021, Yerabham et al., 2018), using the vector pESPRIT002 (Yumerefendi et al., 2010) for transforming *E. coli* BL21 (DE3) pLysE T1R cells. The residue sequences for the constructs are detailed in **Fig. S1**. The culture was grown either in Luria-Bertani (LB) broth or M9 minimal medium. Protein expression was induced at an OD_600_ of 0.6 by the addition of isopropyl-β-D-thiogalactopyranoside (IPTG) to a final concentration of 1 mM and culturing was continued for 16 h at 18 °C. Harvested cells were lysed in Tris-buffered saline (TBS) containing 10 mM Tris-HCl pH 7.4, 150 mM NaCl and Complete EDTA-free protease inhibitor cocktail (Roche) using an ice-chilled microfluidizer M100P (Microfluidics MPT) at 15,000 PSI. The insoluble fraction was removed by centrifugation at 50,000 × g for 25 min at 4 °C. The soluble fraction was loaded onto Co^2+^-loaded NTA resin (Qiagen) and the target protein eluted using TBS containing 500 mM imidazole. The His_6_-C-region protein was further purified using a HiLoad 16/60 Superdex 200 size exclusion chromatography column (GE Healthcare Bio-Sciences).

### Thioflavin-T (ThT) fluorescence assay

The Thioflavin T (ThT) assay was conducted by preparing the reaction mixture immediately before measurement 25 °C on black non-binding 96-well plates (Sigma-Aldrich) with the reaction mixture containing 10 μM ThT and 10 µM protein in a total volume of 100 μL. Experiments were performed in triplicate and each reaction was background-corrected by subtraction of the buffer control. Fluorescence was monitored at regular intervals of 3–5 min using a Fluostar microplate reader (BMG Labtech, Offenburg, Germany) with 440 nm excitation and 492 nm emission filters in bottom-read mode. The signal intensity is depicted as an average value in the form of mean ± SD.

### Dynamic light scattering (DLS)

Measurements of the protein samples were performed using a SpectroSize 300 (XtalConcepts GmbH) instrument with a sample volume of 500 μL at 20 °C. Prior to measurements, all samples were centrifuged at 21,000 × g for 30 min at 4 °C. Diffusion coefficients were obtained from analysis of the decay of the scattered intensity autocorrelation function and were used to determine apparent hydrodynamic radii (R_H_) via the Stokes-Einstein equation, as implemented in the instrument software (SpectroCrystal). Moreover, we determined the detection limit of the DLS instrument as a proxy to the minimal (“critical”) concentration of building blocks supporting fibril growth (see below).

### Isothermal titration calorimetry (ITC)

Measurements were performed using an iTC-200 (MicroCal) calorimeter with 10 µM protein in a 200 µL sample cell. Changes in the heat flow were monitored in real time with the reaction cell stirred at 300 rpm and reference power of the cell set to 5 μcal/s. To compare the thermograms for the different protein, the data set was normalized by dividing each value by the maximum value in the dataset. Furthermore, thermodynamic parameters were computed on the basis of the Goto-Kardos scheme (Kardos et al., 2004, Ikenoue et al., 2014). Assuming that the observed heat exchange represents the enthalpy change of self-association of the DISC1 C-region, ΔH at 20 °C was calculated by integrating the respective peak area. As a framework for describing the thermodynamics of fibrillization, we have previously employed (Cukkemane et al., 2021) a simple 1D “crystallization” model of amyloid formation (Kardos et al., 2004, Ikenoue et al., 2014, Jarrett and Lansbury, 1993); we assume that, upon completion of the process, the protein suspension contains fibrils at equilibrium with residual monomers/oligomers as represented by the “critical concentration” [*C*], which, as a first approximation, equals the reciprocal of the equilibrium association constant *K*_F_. Based on the critical concentration estimated via DLS (Cukkemane et al., 2021, Kardos et al., 2004, Ikenoue et al., 2014, Jarrett and Lansbury, 1993), we obtained the apparent free energy change (Δ*G*_app_ = –*RT*ln*K*_F_ = *RT*ln[*C*], where *R* and *T* are the gas constant and the absolute temperature, respectively). The entropy change Δ*S* was calculated using the values of Δ*H* and Δ*G*_app_ using the Gibbs-Helmholtz equation (Δ*G*_app_ = Δ*H* – TΔ*S*).

### Solid-state NMR experiments (ssNMR)

SsNMR experiments were performed on uniformly ^13^C^15^N- double-labelled samples of the DISC1 C-region and its variants, which were dissolved in TBS buffer containing 1 mM Na_2_-EDTA and 0.01 % (w/v) NaN_3_, and concentrated in a centrifugal device with a 3 kDa cut-off membrane (Amicon, Millipore). The concentrated sample was bath-sonicated for 5 min and incubated at 20 °C for 2 weeks to allow for aggregation. The sample was packed into 3.2 mm zirconia magic angle spinning (MAS) rotors (Bruker Biospin). Measurements were conducted at a static magnetic field strength of 14.1 T (corresponding to a ^1^H Larmor frequency of 600 MHz) using 3.2 mm triple-resonance (^1^H,^13^C,^15^N) Bruker MAS ssNMR probes (Bruker Biospin) at a 12.5 kHz MAS rate. The temperature of the VT gas was set to 256 K, resulting in a sample temperature of 266 ± 5 K. Water-edited 1D build-up experiments were recorded using a ^1^H *T*_2_ filter of 2.5 ms to suppress signals from the rigid regions of the protein. For ^1^H–^1^H spin diffusion, mixing times (*t_m_*) of 2–500 ms were used to permit spin diffusion from water to the protein. ^1^H decoupling was applied during evolution and detection periods using the SPINAL64 (Fung et al., 2000) scheme at a radio-frequency of 83 kHz.

While semi-quantitative, water-edited ssNMR studies provide a reasonable estimate of the molecular dimensions of water accessible areas of membrane proteins (Ader et al., 2009, Luo and Hong, 2010) and amyloid fibrils (Schneider et al., 2011). We implemented a similar approach to calculate and compare the dimensions of WT-C-region fibrils (Cukkemane et al., 2021). Briefly (details in SI methods), the spectral region of 50–75 ppm was integrated and normalized to the maximum signal intensity, which was plotted against the mixing time. In the absence of any saturation effects, the slope describes the time required to reach 100 % magnetization transfer (Ader et al., 2009, Schneider et al., 2011, Schmidt-Rohr et al., 1994). A linear fit to the initial build-up rate ($t_{m}^{s}$) was used to determine the water accessibility of the sample and calculate the molecular dimensions of the fibril. All data were processed and analyzed using Topspin 4.0.6 (Bruker Inc).

### Atomic force microscopy (AFM)

For sample preparation, 5 µL of DISC1 WT C-region, the mutants, or the β-core at concentrations ranging from 1 to 50 µM was applied onto a freshly cleaved muscovite mica surface. Samples were incubated for 10 min under a humid atmosphere, followed by washing thrice with Milli-Q water (100 μL) and finally drying under a stream of N_2_ gas. Images were recorded in intermittent contact mode using a JPK NanoWizard 3 atomic force microscope equipped with a silicon cantilever and tip (OMCL-AC160TS-R3, Olympus). The tip has an average radius of 9 ± 2 nm, a force constant of 26 /Nm and a resonant frequency of approx. 300 kHz. The images were processed using JPK Data Processing Software (version spm-5.0.84). The height profiles in the figures were obtained from measured heights using a polynomial fit that was subtracted from each scan line first independently and then using limited data range.

## Results

### Sensitivity of ThT dye to the C-region mutants

The enhanced and red-shifted fluorescence of ThT upon binding to structures enriched in cross β-sheet is used in standard assays to study amyloidogenic proteins. In the case of human DISC1, one study investigating the recombinant full-length protein noted increases ThT fluorescence, indicating formation of cross β-fibrils (Tanaka et al., 2017). We reported previously (Cukkemane et al., 2021) that fibrils formed by the isolated C-region of the DISC1 protein bind ThT and share structural similarities with other well-characterized neurological proteinopathy markers. However, the aggregates isolated from brain tissue of deceased subjects suffering from schizophrenia, BD and MDD do not stimulate ThT fluorescence (Leliveld et al., 2009, Korth, 2012). To resolve these apparent discrepancies, we investigated the effects of clinically identified alterations S704C, S713E, and L807-FS in ThT fluorescence assays of the DISC1 C-region (Fig. 1**C**). The S713E protein displays ThT fluorescence similar to the WT protein. However, the S704C and L807-FS mutants yielded lower signal intensities. According to our hypothesis, the β-core region represents the lynchpin motif for amyloid-like fibrillization of the DISC1 C-region (Cukkemane et al., 2021). Nonetheless, ThT-reactivity of the β-core construct was minimal with a fluorescence intensity resembling that observed for S704C and L807-FS.

This raises the important question, which structural features within the protein provide the interface for ThT binding and to what extent these traits are modified in the variants investigated. To address these questions, we used a combination of biophysical and structural biology techniques, including DLS, and AFM to comprehend the behavior of the proteins in solution.

### DISC1 C-region aggregates seed fibrillar growth

**Fig. 2:**
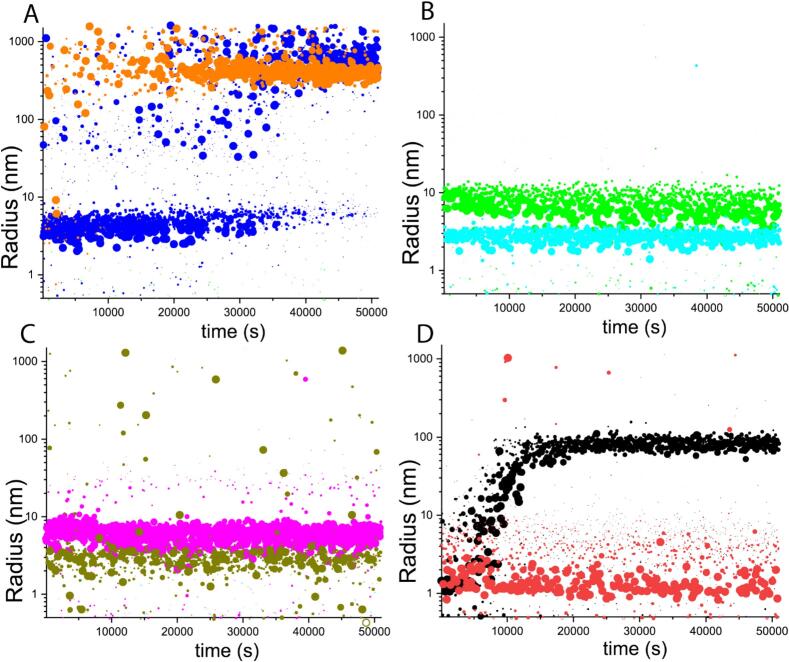
Intensity-weighted DLS isotherms of the DISC1 C-region, mutant variants, and the β-core at 20 °C. (A) MBP-DISC1-C-region (orange) and the remaining constructs carried the his-tag namely WT-C-region (blue); (B) S704C (cyan) and L807-FS (green); (C) two preparations of S713E (magenta and olive green); (D) two samples of the β-core under identical conditions but from two different batches. (For interpretation of the references to colour in this figure legend, the reader is referred to the web version of this article.)

As reported previously (Cukkemane et al., 2021), the WT DISC1 C-region **(**Fig. 2**A**) self-associates into fibrils at 20 °C. Notably, the protein adopts additional multimeric states such as amorphous aggregates at 45 °C and shorter clumped fibrils at 50–60 °C, which do not display ThT fluorescence. In the DLS experiments with C-region variants, at a protein concentration of 10 µM, the major populations of S704C and S713E (Fig. 2**B and 2C**) showed apparent *R*_H_ values of 2.8 ± 0.4 nm and 2.9 ± 0.6 nm, respectively, similar to the WT C-region monomer (2.6 ± 0.5 nm) (Cukkemane et al., 2021). In a couple of situations, the S713E variant did form larger species (*R*_H_ = 6.3 ± 1.2 nm, magenta trace in Fig. 2**C**). The third C-region variant, L807-FS, on average displayed a particle radius of 8.9 ± 1.9 nm (Fig. 2**B**). This is in stark contrast with the behavior of the WT C-region protein, which initially presented as a tetramer (*R*_H_ = 4.25 ± 0.25 nm), but quantitatively transformed into µm range aggregates in the course of the experiment (Fig. 2**A**), in accordance with previous observations (Cukkemane et al., 2021). Unlike the WT C-region, the mutant variants were present in different states of oligomerization/aggregation but did not show an obvious tendency to convert into larger species in a sigmoidal fashion.

**Fig. 3:**
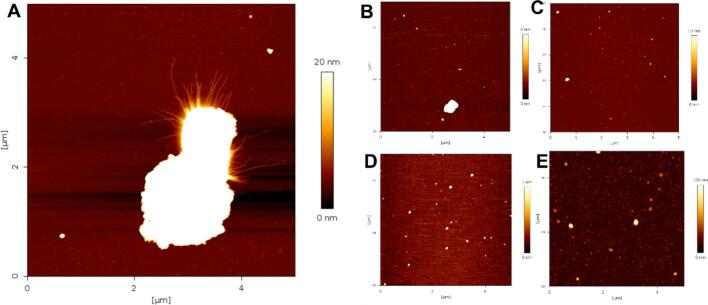
Structural characterization of the DISC1 C-region using AFM (**also see Fig. S2 and S3**). (A) AFM image of fibrillary outgrowth from an aggregate of MBP-C-region. AFM images of the His-tagged DISC1 C-region variants S713E (B), S704C (C), β-core (D) and L807-FS (E) show the presence of aggregates. Protein concentrations were 3 µM (panel A) and 1 µM (panels B-E).

To complement the above findings, we performed a series of AFM (Fig. 3, S2 and S3) measurements to characterize the architecture of the different species observed in the DLS experiments. At protein concentrations ≥ 10 µM (**Fig. S2 and S3**), we consistently observed the presence of numerous amorphous aggregates. Serendipitously, for the 3 µM MBP-C-region protein, we captured an AFM image of a seemingly amorphous aggregate from which fibrils of various lengths were radiating out (Fig. 3**A**). This raises an interesting prospect: Are the amorphous aggregates the seeds for fibrillar growth? Does a higher concentration of the protein (≥ 10 µM) promote amorphous aggregate formation to an extent that does not leave sufficient soluble protein for fibrillar growth? To systematically investigate this effect, we repeated the experiment with a protein concentration of 1 µM (Fig. 3**B-E)**. However, unlike the WT-C-region, in this case we did not observe any fibril formation. Interestingly, in these cases, we observed fewer but larger aggregates than the ones detected with protein concentrations above 10 µM (**Fig. S2**). This indeed indicates that at higher protein concentration, the C-region variants undergo nucleation at an enhanced rate, resulting in the formation of a large number of smaller aggregates because of a rapidly decreasing concentration of free protein, while at lower protein concentrations, one observes slow nucleation yielding larger aggregates. While we do not have enough evidence at the moment, we would like to speculate that these larger aggregates promote the growth of longer fibrillar structures (Cukkemane et al., 2021); Fig. 3**A**) either by directly feeding the growing fibrils or due to the higher concentration of free protein. Our attempts to perform DLS experiments with protein concentrations < 3 µM (**Fig. S4**) did not yield reproducible results because of finite instrument sensitivity.

Next, reverting to one of our major hypotheses, does the β-core represent the scaffold of DISC1 fibrils? In DLS recordings, we observe a sigmoidal aggrandization of the motif. However, unlike the C-region protein that consistently formed larger species at 20 °C, β-core aggregated in ∼ 25 % of trials only (Fig. 2**D**), and the observed aggregates resembled those of the other C-region variants in AFM (Fig. 3**D**) as opposed to the µm long fibrils observed for the WT- and MBP-C-region (Cukkemane et al., 2021); Fig. 3**A**). Together, these findings suggest that the β-core has a tendency to form larger aggregates at lower protein concentrations than the WT-C-region. Based on the DLS experiments we can surmise that it also contains the minimal building block, and that factors like protein concentration and temperature are critical for nucleation and fibril growth. Finally, to sum up our findings on the self-association of the DISC1 C-region variants, we observed the presence of aggregates that were rather heterogeneous in size.

### Thermodynamic underpinning of C-region and variants fibrillization

**Fig. 4:**
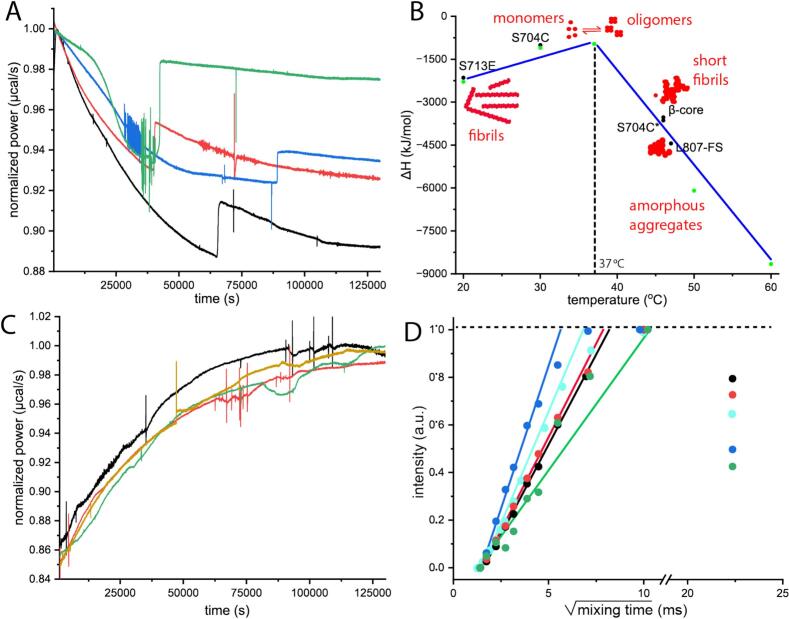
Thermodynamic characterization of the self-association process of the WT C-region, its mutants, and the β-core. (A) The enthalpy changes observed for a 10 µM his-tagged protein solution of WT-C-region at 20 °C (blue), WT-C-region at 30 °C (green), S713E (black), S704C (red). (B) Pictorial representation highlighting the similarities of enthalpy changes between the WT-C-region at various temperatures (green circles) on the one hand and the mutant variants as well as the β-core at 20 °C (black circles) on the other. (C) L807-FS (red), β-core (green), WT-C-region at 50 °C (orange (image adapted from Fig. 3A from Cukkemane et al, 2021 (Cukkemane et al., 2021)), and one-week old S704C (black). (D) Normalized intensities obtained for 1D water-edited (see Fig. S5 for spectra) build-up curves from ^13^C spectra for signals corresponding to Cα region (50–75 ppm) were plotted against the square root of the ^1^H–^1^H mixing time for S713E (blue), S704C (red), L807-FS (green), β-core (black), and the his-tagged WT-C-region (cyan (image adapted from Fig. 3D from Cukkemane et al, 2021 (Cukkemane et al., 2021)),. Straight lines represent the fitted slopes that were used for estimation of $t_{m}^{s}$. (For interpretation of the references to colour in this figure legend, the reader is referred to the web version of this article.)

We were interested in understanding why the mutant proteins and the β-core at 20 °C displayed characteristics that were apparently different from those of the WT-C-region. Thus, we investigated the thermodynamics of aggregation and related our findings to the structure of the assembly. For the WT-C-region, we reported previously (Cukkemane et al., 2021) that fibrils formed at lower temperatures (20–37 °C) were considerably longer than those cultivated at higher temperatures (45–60 °C), and that fibril morphology was related to reaction enthalpy. Intriguingly, the C-region variants investigated at a single temperature were found to divide into two categories simulating the two thermal regimes of the WT protein. Specifically, the S713E and S704C mutants showed an exothermic burst Δ*H* of –2144 kJ/mol and –1004 kJ/mol, respectively, in the course of self-association at 20 °C (Table 1, Fig. 4**A, and 4B**), similar to the WT-C-region at lower temperatures (Δ*H* of –2289 kJ/mol at 20 °C and −1103 kJ/mol at 30 °C; Table 2, **and** Fig. 4**B**). In contrast, the β-core and L807-FS revealed a more profound exothermic aggregation reaction with Δ*H* of −3528 kJ/mol and −4444 kJ/mol, respectively (Table 1**)**, resembling those observed for the WT-C-region at elevated temperatures (Δ*H* of −2761 kJ/mol, −6092 kJ/mol and −8657 kJ/mol at 45, 50 and 60 °C; Fig. 4**B, and 4C**, Table 2 (Cukkemane et al., 2021). However, comparing the enthalpic changes and structural features observed during WT-C-region aggregation at different temperatures with the data obtained for the mutant variants, we observed intriguing similarities: pronounced exothermic reactions correlate with the formation of aggregates but not with the development of long fibrils.

**Table 1:** Thermodynamic properties of the self-association process of the DISC1 C-region mutants and the β-core. The value of ΔG_app_ at 37 °C represents an estimate determined for the WT protein.

C-region variant	ΔH[kJ/mol]	ΔG_app_[kJ/mol]
S704C	−1004	−34
S713E	−2144	
L807-FS	−4444	
β-core	−3528	
S704C*	−3635	

*One week old sample.

**Table 2:** Comparison of enthalpy changes recorded for C-region mutant variants and the β-core with those of the WT-C-region at various temperatures (20–60 °C).

C-region variant	ΔH [kJ/mol]	WT-C-region (Temperature ^o^C)	ΔH[kJ/mol]
S713E	−2144	20	−2289
S704C	−1004	30	−1103
β-core	−3528	37	−962
S704C *	−3635	45	−2761
L807-FS	−4444	50	−6092
		60	−8658

*One week old sample.

Based on the estimated critical concentration of 0.75 µM at 20 °C (Cukkemane et al., 2021), we calculated the ΔG_app_ for fibrillization of the WT-C-region to be −34 kJ/mol. Assuming the overall free energy change is not altered dramatically in the variants investigated in this study, the widely varying reaction enthalpies determined experimentally would still translate into negative entropy changes (between −3.5 and −15 kJ/mol/K) indicating the assembly of highly ordered structures possibly incorporating ordered water molecules. We performed water-edited ssNMR experiments to investigate the mechanism of fibrillization in a qualitative fashion and to understand the molecular structure of the aggregates. In such a study, polarization from water is transferred from the interfacial region to the protein, where a short transfer time ($t_{m}^{s}$) is indicative of high degree of water accessibility for a large part of the protein.

On performing the 1D water build-up experiment (**Fig. S5 and** Fig. 4**D)** and analyzing signals in the region of 50−75 ppm that represent primarily backbone Cα nuclei, we observed tightly bound water, with average initial $t_{m}^{s}$ values of 31.14, 39.21, 62.50 and 34.36 ms (Table S1) for S704C, S731E, L807-FS, and β-core variants, respectively. In comparison the WT-C-region has $t_{m}^{s}$ of 45 ms (Cukkemane et al., 2021), and previous studies on Aβ (Dregni et al., 2020) have shown that water which associated with fibers, presented in interfibrillar spaces and in the bulk have relaxation times of 90, 150, and 250, respectively. This observation supports the notion that water molecules are in proximity to the protein surface for all the constructs.

**Fig. 5:**
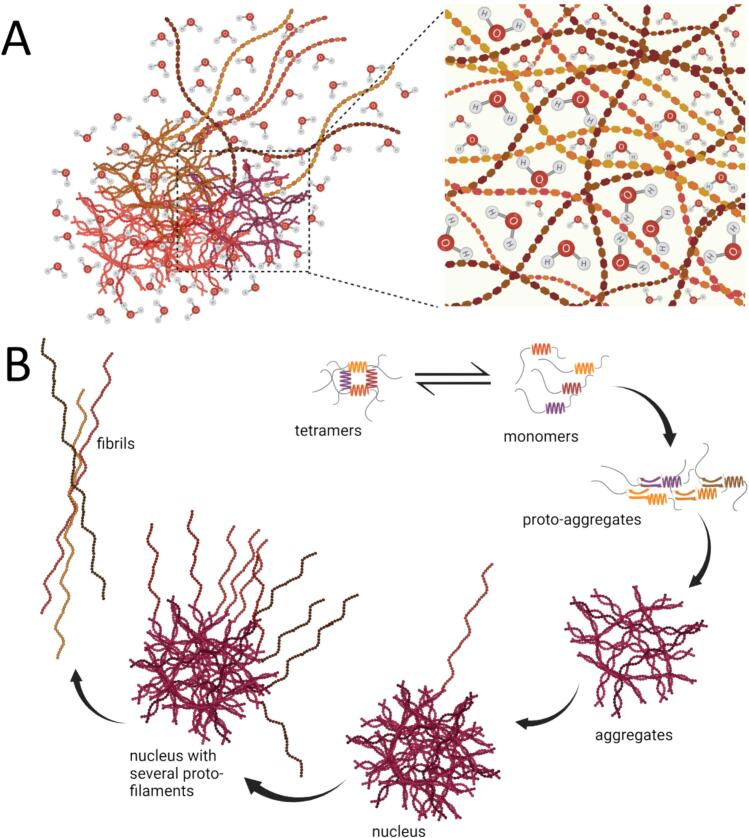
(A) Presumptive model of a DISC1 C-region aggregate including water molecules filling cavities and associated along the length of the fibrils. (B) Snapshots describing the aggregation and fibrillar pathway of the DISC1 C-region; the mutant variants are suggested to follow a similar pathway.

However, to understand the finer details of the aggregates observed in AFM images (Fig. 3 and **Fig. S3**), we were curious to understand their molecular organization. We hypothesized that the aggregates are composed of a mesh of filaments that serves as a seed for fibrillar growth. Therefore, we assumed a structural model of an elongated fibril with a circular cross-sectional area (Schmidt-Rohr et al., 1994); based on this, we calculated the diameters (supplementary methodology) of the different fibrils to be 5.73, 5.56, 15.92, and 5.92 nm (**Table S1**) for S704C, S713E, L807-FS, and β-core, respectively. Barring the L807-FS mutant, these fibril diameters would be comparable to those of the WT construct (5−7 nm (Cukkemane et al., 2021). But unlike the spectra for other C-region constructs and the β-core, the L807-FS shows a noisy spectrum due to protein instability during preparation and lesser sample in the NMR rotor. However, comparing the buildup time of L807-FS (62.50 ms) with those reported for other amyloid fibrils, which have $t_{m}^{s}$ of 17–110 ms (Schneider et al., 2011, Dregni et al., 2020, Wang et al., 2017), for ordered water molecules interacting with the core region of β-fibrils, the data suggest that the aggregates of L807-FS may contain an underlying fibrillar assembly. The findings suggest that the seemingly amorphous aggregates may constitute a mesh-like network that contains water molecules associated within the structure and some bound on the surface (Fig. 5**A**). However, with the information we have acquired, we believe that the aggregated material acts as the seed that promotes the assembly of the larger structures observed in the DLS and AFM analyses.

## Discussion

The aim of this study has been to further structurally characterize the DISC1 C-region and to understand its significance to the pathophysiology of CMIs such as schizophrenia, MDD and BD. Following this line, we characterized three mutants (Fig. 1**A and 1B**) of the C-region using a combination of biophysical and structural biology applications. Intuitively, one would expect that the mutation in the S713E C-region variant will not have a dramatic effect on protein stability, while the impact of the Ser-to-Cys exchange in position 704 is more difficult to predict. In line with this expectation, S713E displays ThT fluorescence similar to that of the WT-C-region (Fig. 1**C**), along with a similar thermogram at 20 °C (Fig. 3**B**). In contrast, S704C shows weak ThT reactivity (Fig. 1**C**) and its thermogram is reminiscent of the WT-C-region at 30 °C. We also tested a one-week-old S704C sample for its thermodynamic properties; in this case, the thermogram at 20 °C resembled the profile of the WT-C-region at 50 °C (Fig. 4**B** and Table 2). Our findings on the S704C C-region may therefore hint at a heterogeneous nature of the aggregates in post-mortem brain samples (Leliveld et al., 2009, Tanaka et al., 2017) and their inability to bind to the ThT dye. To summarize our observations, the mutations where a polar Ser is exchanged to Glu/Cys are tolerated but the behavior and the stability of the resulting proteins are reminiscent of the WT-C-region maintained at different temperatures. In comparison with the mutant variants mentioned above, L807-FS also showed low intensity ThT fluorescence (Fig. 1**C**), but it rapidly aggregated and was very unstable in solution. The L807-FS mutant displayed a calorimetric footprint that resembled the WT protein at 50 °C.

As an extension of our previous study on the DISC1 C-region fibrillization process (Cukkemane et al., 2021), we wanted to comprehend the role of the pseudo-repeat sequence (717−761), i.e., the β-core. In accordance with our hypothesis, the β-core displayed a sigmoidal trend of increase in particle size like the WT-C-region, with amorphous aggregates being the prevalent species under all tested conditions. These findings are of strong physiological significance because it includes half of the amino acid stretch of the Δ22 region (748–769, (Kamiya et al., 2006), which is absent in DISC1^Δ22aa^. When expressed in PC12 cells, DISC1^Δ22aa^ is incapable of interacting with NUDEL and LIS1 from the mitotic complex, which prohibits the proliferation of the cell line. In a similar vein, we believe that the participation of the β-core in fibrillar assembly will impair DISC1 function, presumably hampering mitosis and ensuing processes such as cell division, neuronal proliferation, and migration.

Intriguingly, the ThT fluorescence signal arising from this dye binding to DISC1 has been reported only in a study evaluating DISC1 function in relation to Hungtinton’s disease (Tanaka et al., 2017), where the presence of DISC1 as fibers was demonstrated, and in our previous report (Cukkemane et al., 2021), where we showed that the fibers of the C-region were β-fibrils. This raises the important question why DISC1 reactivity with ThT has been observed only sporadically. At least part of the explanation may be related to the heterogeneous nature of the aggregates, which is corroborated by the coexistence of (seemingly) amorphous material and fibrils, which is concentration and temperature dependent (Cukkemane et al., 2021) (Fig. 3, S2-S3). In this context, it is also important to highlight that binding of fluorescent dyes such as ThT and Congo red occurs in the fibrillar grooves, which are formed by multiple protofilaments (Schutz et al., 2015, Frieg et al., 2020). Such a process involves the intercalation of ThT between two filaments. Until now, however, all fibrils of the DISC1 C-region (Cukkemane et al., 2021)(Fig. 3**A**), that have been observed appear as mono-filamentous assemblies. This may underlie the observed poor fluorescence of ThT in the presence of the WT-C-region (Cukkemane et al., 2021) as well as the recombinantly expressed mutants; extrapolating to the full-length protein, these considerations would also explain the absence of fluorescent signals from samples obtained from post-mortem brain tissues (Leliveld et al., 2009, Korth, 2012).

So, what drives the DISC1 C-region into an amyloid-like structure and what similarities does it share with its counterparts from the neurodegenerative world? From a thermodynamic perspective, protein association can often be described by entropy-enthalpy compensation. In such a scenario, formation of aggregates and fibrils is driven by enthalpy loss (Kardos et al., 2004, Ikenoue et al., 2014), while concomitantly the entropy of the system (with protein and solvent contributions *S*_prot_ and *S*_sol_, respectively) is reduced. For all tested samples, a strong exothermic reaction and presence of water was observed. The common feature among amyloids that likely drives aggregation and fibrillar assembly is the accumulation of several weak interactions within the core of the protein involving hydrophobic residues (Gazit, 2002) in repeats (Schneider et al., 2011, Gazit, 2002, Cherny et al., 2005) and pseudo-repeats (Wasmer et al., 2008, Fitzpatrick et al., 2017), which is reflected by enthalpic changes. In several natively disordered and amyloidogenic proteins (Kardos et al., 2004, Ikenoue et al., 2014, Lawrence et al., 2014, Rezaei et al., 2002, Ikenoue et al., 2014, Kinoshita et al., 2018, Jeppesen et al., 2010) a highly exothermic process was reported, potentially highlighting the association of the hydrophobic core. For many amyloids, it has been indicated that water is aligned along the length of the fibril (Schneider et al., 2011, Dregni et al., 2020, Wang et al., 2017). The ordered water may also be arranged in small pockets within the hydrophobic core (Dregni et al., 2020) or, in other examples, be associated as compartments in different pools (Wang et al., 2017) such as surface-bound water (loosely or tightly bound), interfibrillar water and bulk-like matrix water. Altogether, water molecules arranged in a well-ordered fibrillar system will be an indirect indicator of low protein entropy. And a more accurate description of the molecular architecture of the aggregates can be obtained from a combination of high-resolution spectra, D_2_O exchange experiments (Soares et al., 2011), and residue-specific water-edited experiments (Wang et al., 2017).

What exactly constitutes the supra-molecular structure of the DISC1 C-region? The DISC1 protein (Fig. 1**B**) is composed of largely unstructured and four distinct structured regions (Yerabham et al., 2013, Yerabham et al., 2017, Levin et al., 2014). The C-region, which is one of the structured segments is composed of helical coiled-coils (Ye et al., 2017, Wang et al., 2019) and unstructured elements (Cukkemane et al., 2021, Ye et al., 2017), especially in the absence of an interacting partner. The partially disordered and coiled-coil regions provide ample plasticity for integration into various cellular signaling pathways, interacting with homo- or hetero-oligomeric partners. We demonstrated previously (Cukkemane et al., 2021) that the DISC1 C-region has a strong propensity to form tetrameric structures, aggregates, and fibrils. The reaction may largely be driven by hydrophobic interactions (Fig. 1**A**) among residues in the pseudo-repeat of the β-core. Perhaps, the aggregation mechanism potentially progresses via liquid phase separation (Banach et al., 2019, Patel et al., 2015, Fandrich et al., 2018).

These amorphous-like aggregates are likely composed of a mesh-like protein network with water molecules embedded within the structure (Fig. 5**A**), which then serves as a seed for fibrillar growth. We tend to favor the model describing the liquid droplets of the RNA-binding protein FUS (Fandrich et al., 2018), which is a risk factor for amyotrophic lateral sclerosis (ALS). Similar to FUS, we observe that the aggregates (Fig. 3) of the WT C-region serve as seeds and that fibrils extend from such supra-molecular structures. Assuming the C-region and its S704C and S713E mutants share the same pathway to fibrillization, we provide a model with pictorial snapshots (Fig. 5**B)** depicting the growth of the assembly, where the building block is the tetramer, which progressively self-associates to form larger structures; this in turn promotes seeding followed by elongation of the fibrils, which are either short or long depending on the conditions, e.g., temperature.

Taking into account the results presented herein, we presume the conformational flexibility of the C-region and perhaps also that of the full-length DISC1 may be key to enabling interactions with a myriad of partners (Yerabham et al., 2013, Millar et al., 2003) as a scaffold protein. At the same time, this plasticity likely facilitates the formation of heterogenous aggregates (Fig. 3, S2 and S3) in DISC1opathies (Korth, 2012), which is relevant to the clinical findings from human brain tissue. For this reason, we strongly advocate a structure-based rationale in drug design. This is because the heterogeneous nature of the particles including the sporadic presence of fibrils has posed a significant challenge to the drug development strategy aiming to prevent aggregation in neurodegenerative disorders (Plascencia-Villa and Perry, 2023). As an example of recent relevance, several reasons have been put forward for the relatively poor efficacy of the drug mAb Aducanumab directed against aggregated Aβ (Wojtunik-Kulesza et al., 2023), including structural implications (Colvin et al., 2016). It has been argued that the mAb binds to an N-terminal region that is unstructured (Gremer et al., 2017) or structured (Cukkemane and Willbold, 2022) under different conditions, thereby limiting the effectiveness of the drug. Taking a leaf out of the lessons with neurodegenerative disease targets and avoiding similar pitfalls, we have implemented a different approach. We are developing peptide therapeutics based on a hit-and-lead strategy using the phage-display technique [57], explicitly targeting either monomers or oligomers, which are generated using the conditions described herein and reported previously (Cukkemane et al., 2021). The aim is to identify peptides which bind individual protomers in the aggregate or fibril, thereby uncoupling them from the large supramolecular assembly and restoring DISC1 function. Clearly, DISC1 is involved in a variety of physiological processes, interacts with many potential partners, and may also have overwhelming structural diversity. Therefore, it is pertinent to further characterize structure and function of the different domains of this large scaffold protein when rationalizing an adequate drug development strategy.

## Authors contribution

AC, DW, and OHW designed the experiments and drafted the manuscript. AC prepared all the samples and performed experiments using DLS and ITC. ssNMR experiments were performed by AC alongside NB and HH. AFM was performed by TK. All authors critically reviewed the manuscript.

## CRediT authorship contribution statement

**Abhishek Cukkemane:** Writing – review & editing, Writing – original draft, Visualization, Validation, Supervision, Resources, Project administration, Methodology, Investigation, Formal analysis, Conceptualization. **Nina Becker:** Writing – review & editing, Methodology, Investigation. **Tatsiana Kupreichyk:** Writing – review & editing, Methodology, Investigation. **Henrike Heise:** Writing – review & editing, Resources, Methodology, Investigation, Funding acquisition. **Dieter Willbold:** Writing – review & editing, Writing – original draft, Supervision, Resources, Project administration, Funding acquisition, Conceptualization. **Oliver H. Weiergräber:** Writing – review & editing, Writing – original draft, Supervision, Resources, Project administration, Funding acquisition, Conceptualization.

## Declaration of competing interest

The authors declare the following financial interests/personal relationships which may be considered as potential competing interests: AC and DW are inventors of patents covering the peptide drugs. All other authors declare no competing interests

## Data Availability

No data was used for the research described in the article.
