# Supplementary material for: Tracing the aggregation pathway of the scaffold protein DISC1: Structural implications for chronic mental illnesses
Source: J Struct Biol X. 2025 May 24;11:100128. doi: 10.1016/j.yjsbx.2025.100128 (PMC12158487; doi:10.1016/j.yjsbx.2025.100128)
Supplement: Supplementary Data 1 [file mmc1.docx]

**SUPPLEMENTARY INFORMATION**

**Tracing the aggregation pathway of the scaffold protein DISC1: structural implications for chronic mental illnesses**

Abhishek Cukkemane^1,2^*, Nina Becker^1,2^, Tatsiana Kupreichyk^1,2^, Henrike Heise^1,2^, Dieter Willbold^1,2^* and Oliver H. Weiergräber^1^*

^1^Institute of Biological Information Processing (IBI-7: Structural Biochemistry), Forschungszentrum Jülich, Jülich, Germany.

^2^Heinrich Heine University Düsseldorf, Institut für Physikalische Biologie, Düsseldorf, Germany.

*email – [a.cukkemane@fz-juelich.de](mailto:a.cukkemane@fz-juelich.de); [d.willbold@fz-juelich.de](mailto:d.willbold@fz-juelich.de); [o.h.weiergraeber@fz‑juelich.de](mailto:o.h.weiergraeber@fzjuelich.de)

**Supplementary methods**

Analysis of the water-edited 1D build-up experiments was performed by integrating the spectral region of 50-75 ppm, followed by normalization to the maximum signal intensity (at 100 ms mixing time). A linear fit to the initial build-up rate $\frac{1}{s}=(t_{m}^{s}$) was used to determine the water accessibility of the sample. The slope (s) describes the time that is required to reach 100% magnetization transfer in the absence of any saturation effects [1], which is inversely proportional to the volume-to-surface area ratio (V/S) described by $\frac{V}{S}=\sqrt{\frac{D_{eff}t_{m}^{s}}{\pi}}$ where *D_eff_* describes the effective magnetization diffusion coefficient corresponding to 0.2 nm^2^/ms [2, 3]. Assuming that the fibril represents an elongated cylinder where the fibrillary length greatly exceeds the diameter (*d*) of the fibril, the ratio of *V/S* equals *d*/4. Such an approach provides a semi‑quantitative estimate of the molecular dimensions of water-accessible areas of membrane proteins [2, 4] and amyloid fibrils [3] in comparison to their low-resolution structural models. Using a similar approach previously, we determined the diameter of the fibrils of the WT C‑region to be 6.8 nm, that corresponds well with the EM findings, which was in the range of 5-7 nm [5].

Table S1: Water-edited buildup data for the C-region constructs investigated. The initial rate slope was obtained from a linear fit to the data points of the buildup curve. Also see Fig. S3 and Fig. 4

| C-region variant | Initial rate slope (1/ms) | $t_{m}^{s}$ (ms) | V/S (nm) | d (nm) |
| --- | --- | --- | --- | --- |
| S713E | 0.033 | 30.30 | 1.39 | 5.56 |
| S704C | 0.031 | 32.26 | 1.43 | 5.73 |
| L807-FS | 0.016 | 62.50 | 3.98 | 15.92 |
| β-core | 0.029 | 34.48 | 1.48 | 5.92 |

**SUPPLEMENTARY FIGURES**

**WT-C-region (MBP-fusion construct)**

MGVHSSHHHHHHSSENLYFQSRTKIEEGKLVIWINGDKGYNGLAEVGKKFEKDTGIKVTVEHPDKLEEKFPQVAATGDGPDIIFWAHDRFGGYAQSGLLAEITPAAAFQDKLYPFTWDAVRYNGKLIAYPIAVEALSLIYNKDLLPNPPKTWEEIPALDKELKAKGKSALMFNLQEPYFTWPLIAADGGYAFKYAAGKYDIKDVGVDNAGAKAGLTFLVDLIKNKHMNADTDYSIAEHAFNHGETAMTINGPWAWSNIDTSAVNYGVTVLPTFKGQPSKPFVGVLSAGINAASPNKELAKEFLENYLLTDEGLEAVNKDKPLGAVALKSYEEELVKDPRVAATMENAQKGEIMPNIPQMSAFWYAVRTAVINAASGRQTVDAALAAAQTNHMGGGSGGGSASLEVLFQGPTSELWEADLEACRLLIQSLQLQEARGSLSVEDERQMDDLEGAAPPIPPRLHSEDKRKTPLKVLEEWKTHLIPSLHCAGGEQKEESYILSAELGEKCEDIGKKLLYLEDQLHTAIHSHDEDLIQSLRRELQMVKETLQAMILQLQPAKEAG

**WT-C-region**

MGHHHHHHDYDIPTTENLYFQGWEADLEACRLLIQSLQLQEARGSLSVEDERQMDDLEGAAPPIPPRLHSEDKRKTPLKVLEEWKTHLIPSLHCAGGEQKEESYILSAELGEKCEDIGKKLLYLEDQLHTAIHSHDEDLIQSLRRELQMVKETLQAMILQLQPAKEAGSG

**S704C**

MGHHHHHHDYDIPTTENLYFQGWEADLEACRLLIQCLQLQEARGSLSVEDERQMDDLEGAAPPIPPRLHSEDKRKTPLKVLEEWKTHLIPSLHCAGGEQKEESYILSAELGEKCEDIGKKLLYLEDQLHTAIHSHDEDLIQSLRRELQMVKETLQAMILQLQPAKEAGSG

**S713E**

MGHHHHHHDYDIPTTENLYFQGWEADLEACRLLIQSLQLQEARGELSVEDERQMDDLEGAAPPIPPRLHSEDKRKTPLKVLEEWKTHLIPSLHCAGGEQKEESYILSAELGEKCEDIGKKLLYLEDQLHTAIHSHDEDLIQSLRRELQMVKETLQAMILQLQPAKEAGSG

**L807FS**

MGHHHHHHDYDIPTTENLYFQGRLVWEADLEACRLLIQSLQLQEARGSLSVEDERQMDDLEGAAPPIPPRLHSEDKRKTPLKVLEEWKTHLIPSLHCAGGEQKEESYILSAELGEKCEDIGKKLLYLEDQLHTAIHSHDEDLSLSGGSSRWSG

**β-core**

MGHHHHHHDYDIPTTENLYFQGDERQMDDLEGAAPPIPPRLHSEDKRKTPLKVLEEWKTHLIPSLHCAGGEQKEESSG

Fig S1: The amino acid sequences of the DISC1 WT-C-region protein constructs, S704C, S713E, L807 frame-shift and the β-core


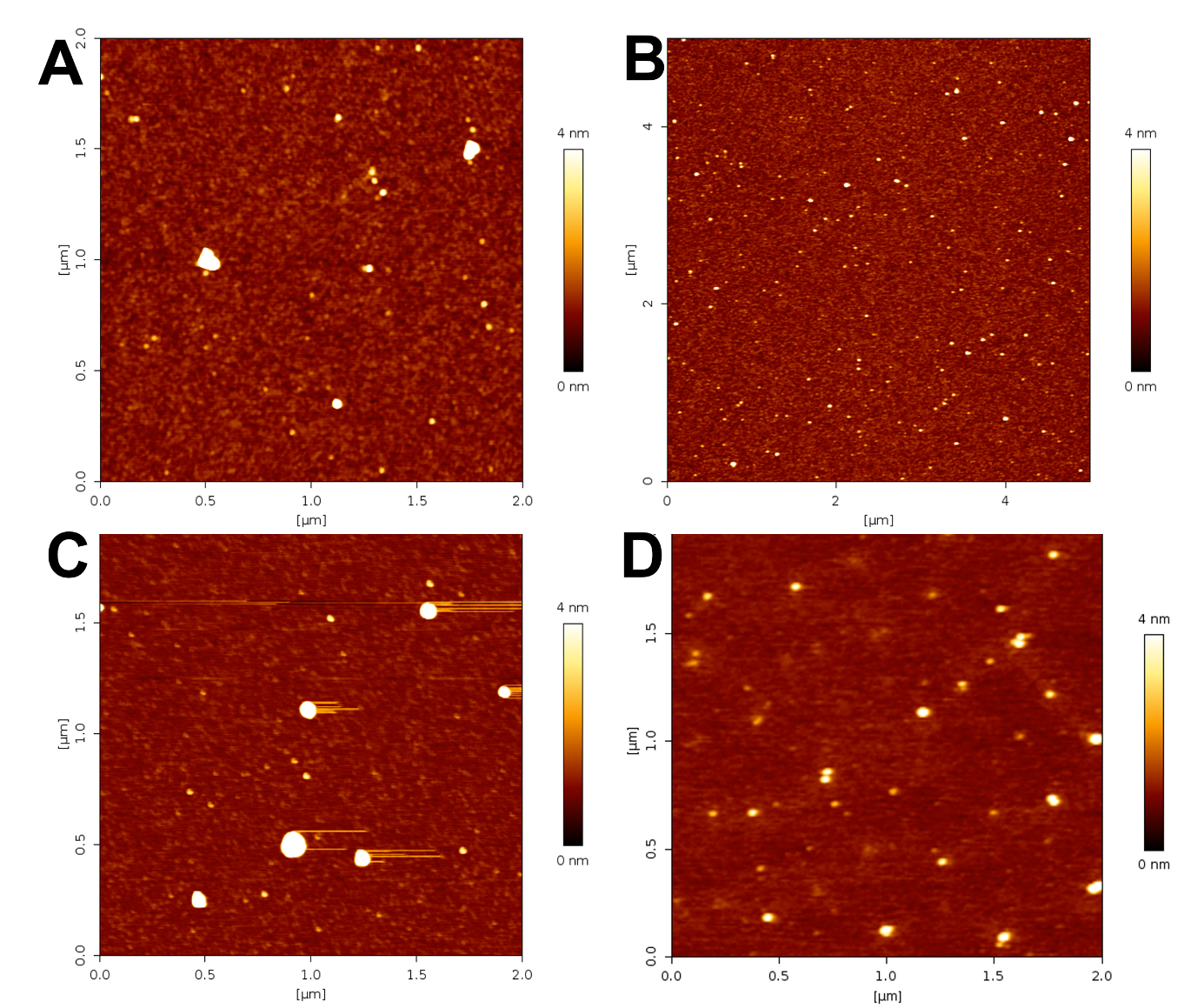


Fig. S2: Images of the His-tagged DISC1 C‑region variants and the β-core at concentrations of 10 µM in panels were recorded using atomic force microscopy (AFM) for (A) S713E, (B) S704C, (C) β-core, and L807-FS.


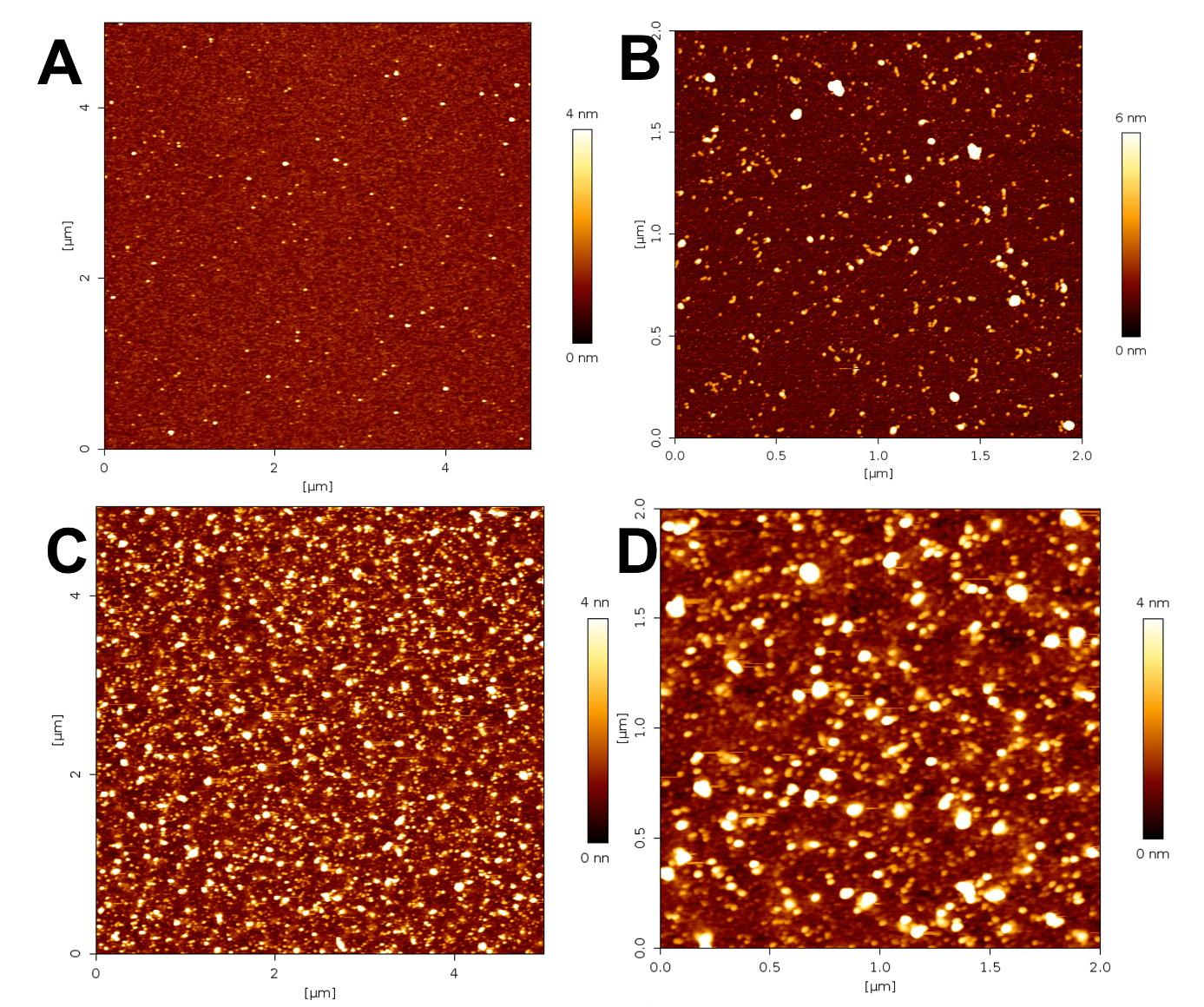


Fig. S3: Images of the His-tagged DISC1 C‑region variants and the β-core at concentrations of 50 µM in panels were recorded using atomic force microscopy (AFM) for (A) S713E, (B) S704C, (C) β-core, and (D) L807-FS.


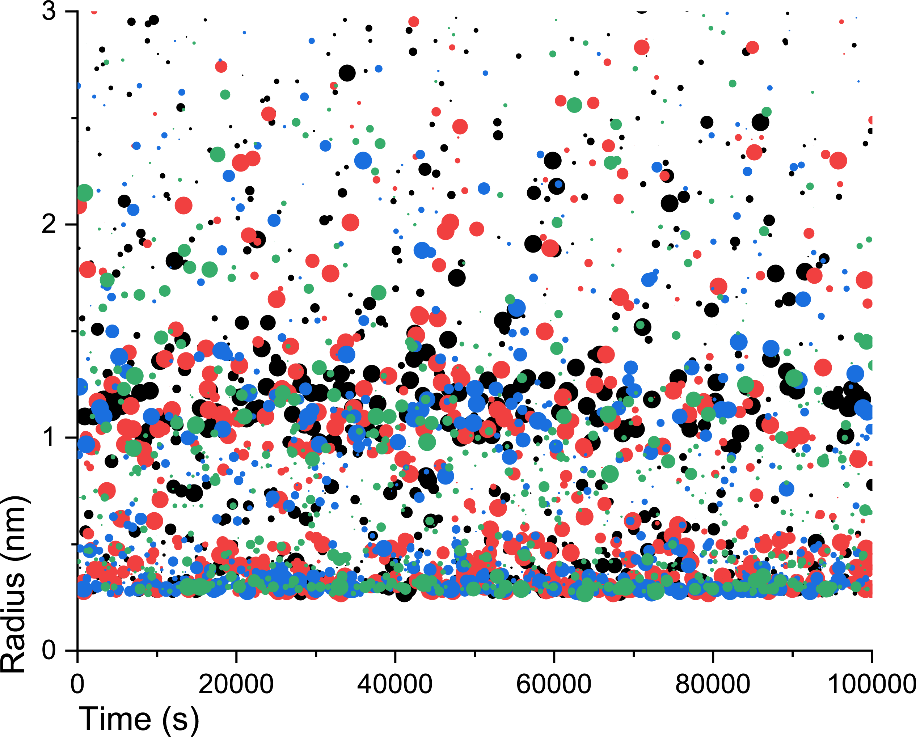


Fig. S4: The figure describes the DLS results of C-region variants at a concentration of 2.5 µM for S704C (red), S713E (black), L807-FS (blue), and β-core (green) at 20 °C.


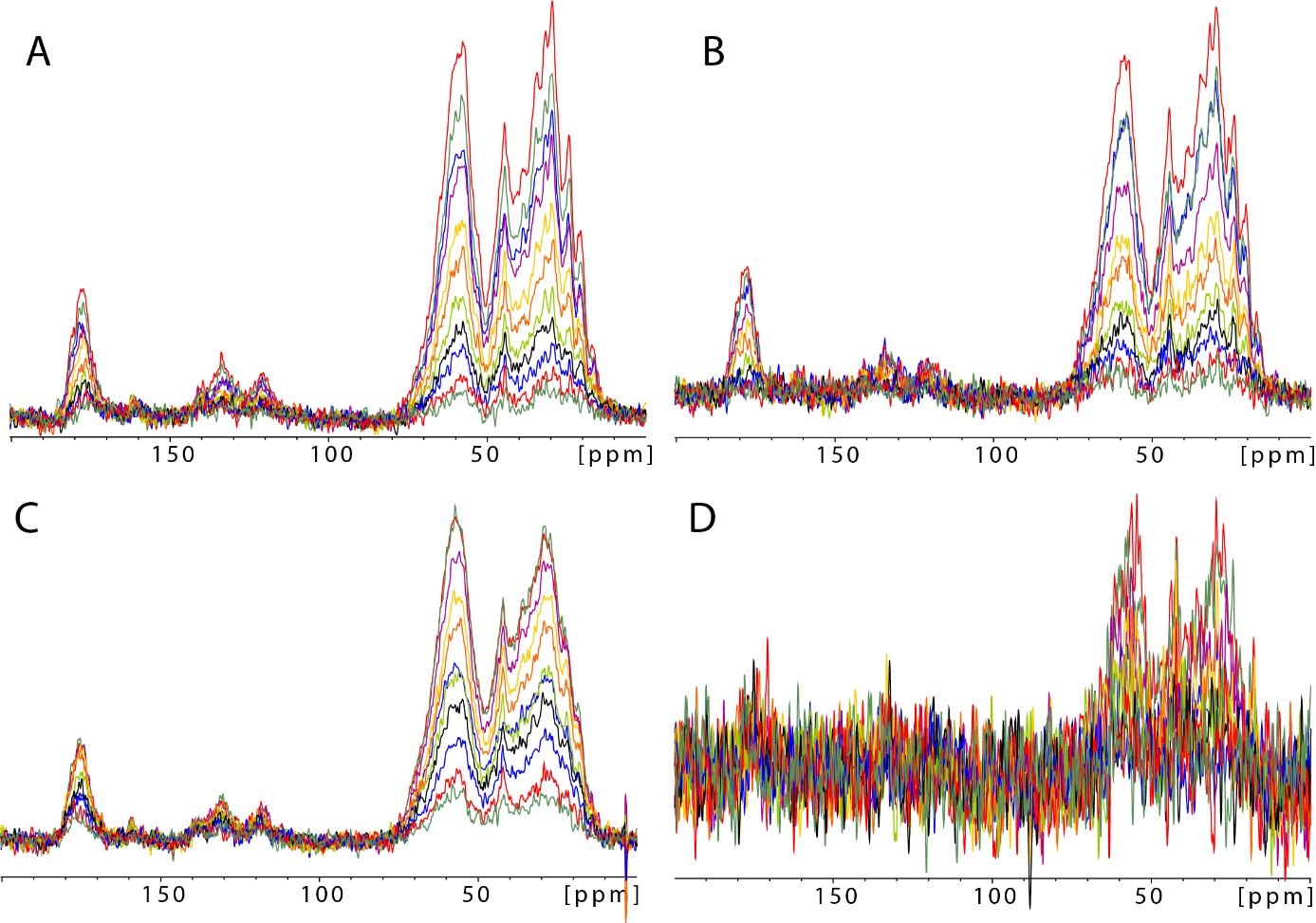


Fig. S5: 1D ^13^C water build-up spectra of (A) S713E, (B) S704C, (C) β-core, and (D) L807-FS with several ^1^H-^1^H mixing times ranging from 500 ms (blue), 100 ms (red), 50 ms (green), 30 ms (magenta), 20 ms (yellow), 15 ms (orange), 10 ms (light-green), 7 ms (black), 5 ms (blue), 3 ms (red), and 2 ms (green), respectively.

**References:**

1. Schmidt-Rohr K, Spiess HW: **CHAPTER ONE - Introduction**. In: *Multidimensional Solid-State NMR and Polymers.* edn. Edited by Schmidt-Rohr K, Spiess HW. San Diego: Academic Press; 1994: 1-12.

2. Ader C, Schneider R, Seidel K, Etzkorn M, Becker S, Baldus M: **Structural rearrangements of membrane proteins probed by water-edited solid-state NMR spectroscopy**. *J Am Chem Soc* 2009, **131**(1):170-176.

3. Schneider R, Schumacher MC, Mueller H, Nand D, Klaukien V, Heise H, Riedel D, Wolf G, Behrmann E, Raunser S *et al*: **Structural characterization of polyglutamine fibrils by solid-state NMR spectroscopy**. *J Mol Biol* 2011, **412**(1):121-136.

4. Luo W, Hong M: **Conformational changes of an ion channel detected through water-protein interactions using solid-state NMR spectroscopy**. *J Am Chem Soc* 2010, **132**(7):2378-2384.

5. Cukkemane A, Becker N, Zielinski M, Frieg B, Lakomek NA, Heise H, Schroder GF, Willbold D, Weiergraber OH: **Conformational heterogeneity coupled with beta-fibril formation of a scaffold protein involved in chronic mental illnesses**. *Transl Psychiatry* 2021, **11**(1):639.
